# Comparison of response rates and cost-effectiveness for a community-based survey: postal, internet and telephone modes with generic or personalised recruitment approaches

**DOI:** 10.1186/1471-2288-12-132

**Published:** 2012-08-31

**Authors:** Martha Sinclair, Joanne O’Toole, Manori Malawaraarachchi, Karin Leder

**Affiliations:** 1 Department of Epidemiology and Preventive Medicine, School of Public Health and Preventive Medicine, Monash University, Melbourne, Australia; 2 School of Land and Environment, The University of Melbourne, Parkville, Australia

**Keywords:** Survey methods, Postal survey, Telephone survey, Internet survey, Cost-effectiveness

## Abstract

**Background:**

Epidemiological research often requires collection of data from a representative sample of the community or recruitment of specific groups through broad community approaches. The population coverage of traditional survey methods such as mail-outs to residential addresses, and telephone contact via public directories or random-digit-dialing is declining and survey response rates are falling. There is a need to explore new sampling frames and consider multiple response modes including those offered by changes in telecommunications and internet technology.

**Methods:**

We evaluated response rates and cost-effectiveness for three modes of survey administration (postal invitation/postal survey, postal invitation/internet survey and postal invitation/telephone survey) and two styles of contact approach (personalised and generic) in a community survey of greywater use. Potential respondents were contacted only once, with no follow up of non-responders.

**Results:**

The telephone survey produced the highest adjusted response rate (30.2%), followed by the personalised postal survey (10.5%), generic postal survey (7.5%) and then the internet survey (4.7% for the personalised approach and 2.2% for the generic approach). There were some differences in household characteristics and greywater use rates between respondents to different survey modes, and between respondents to personalised and generic approaches. These may be attributable to the differing levels of motivations needed for a response, and varying levels of interest in the survey topic among greywater users and non-users. The generic postal survey had the lowest costs per valid survey received (Australian $22.93), followed by the personalised postal survey ($24.75).

**Conclusions:**

Our findings suggest that postal surveys currently remain the most economic option for population-based studies, with similar costs for personalised and generic approaches. Internet surveys may be effective for specialised groups where email lists are available for initial contact, but barriers other than household internet access still exist for community-based surveys. Given the increasing recruitment challenges facing community-based studies, there is an imperative to gather contemporary comparative data on different survey modes and recruitment approaches in order to determine their strengths, limitations and costs. Researchers also need to document and report on the potential biases in the target and respondent populations and how this may affect the data collected.

## Background

Modes of contact for community-based epidemiological research traditionally include mail-outs to residential addresses, and telephone contact based on public directories or random-digit-dialing (RDD) to landline telephone numbers. In addition to deciding on contact mode(s), a choice needs to be made between personalised (directed to a named person) or generic (to the Householder/Occupant) approaches. However, the population coverage of personalised data sources based on public telephone directory listings has declined in recent years due to the rapid transition to wireless (mobile/cell) phones [1,2], and higher proportions of unlisted landline numbers [3]. The rising prevalence of households with only wireless telephone connections has also impacted adversely on the coverage of generic RDD methodology for landline telephone numbers [2].

In addition, researchers are encountering greater problems in making contact with potential respondents and rising refusal rates among those contacted [4]. Declining response rates have been reported for long established population health surveys including the U.S. Behavioral Risk Factor Surveillance Study (BRFSS), as well as public opinion and consumer surveys [4]. A review of the RDD monthly Survey of Consumer Attitudes by the University of Michigan [5] showed non-contacts accounted for less than 5% of non-response between 1979 and 1985, but increased to more than 15% by 2003. The rate of refusals among contacted households also increased from 19% in 1979 to 27% in 2003, and the overall response rate fell from 72% to 48%. The dual challenges of shrinking population coverage of traditional sampling frames and falling response rates underline the need to explore new sampling frames and alternative modes of data collection for community-based surveys and to document their utility.

### Use of the internet for community surveys

With internet access now widespread in many countries, electronic methods of contact and data gathering for surveys have become possible. In common with telephone interviews, internet surveys offer many advantages for improving data quality compared to written questionnaires [6]. These include the ability to require or prompt for answers to missed questions, application of input masks and consistency checks to minimise invalid responses, and skipping of irrelevant sections which are conditional on the answers to previous questions. Problematic questions can be quickly identified by tracking completion times and break-off points, then modified to reduce the rate of incomplete surveys [7]. Initial costs for internet surveys vary depending on the level of programming sophistication required, but as data entry is performed by the respondents, the cost per response declines as the number of respondents increases. This offers potential savings compared to postal or telephone survey modes where material and staff costs tend to be proportional to respondent numbers.

For surveys of specialised populations such as students or professional groups it is usually possible to send an email invitation with a direct hyperlink to the internet survey to an existing email list, but the lack of such lists for the general population means that other methods of contact need to be used. Comparisons of internet surveys with other modes of survey administration have generally used conventional mail for initial contact, and a common finding has been that response rates to community internet surveys are lower than for postal surveys [8-10]. Two independent meta–analyses found that the average differential between internet surveys and other response modes was greater for community surveys than for surveys involving specific target groups such as students, employees or association members who may be more familiar with internet use [11,12].

Even in countries such as Sweden where more than 80% of adults have internet access at home and there is high penetration across different age groups and education levels [9], it appears most people still prefer mail surveys. For example, a community internet health survey with one mail and one telephone follow up contact following a mailed invitation, obtained a response rate of 50.6%, but the parallel postal survey arm yielded a response rate of 64.4% [13]. Similarly, a lifestyle survey of Swedish women produced an initial response rate of 33% for a postal invitation to complete an internet survey [14], but when non-respondents were followed up by letter or email and offered the choice of postal or internet response, over 90% of those who responded at this stage chose the postal option.

Although internet surveys currently appear less effective for community studies than other response modes, the combination of multiple modes may offer a means to improve overall survey response rates and possibly broaden population coverage [15]. Indeed, it has been argued that use of any single mode of contact other than face-to-face household interviews will currently exclude significant sub-groups within the population [16]. Studies offering the internet mode simultaneously with other response modes have generally found little improvement in overall response rates [17,18], but sequential use of different modes has been reported to be beneficial [15]. For example, a US community study using a generic address-based postal sampling frame found that response rates between 44% and 52% could be achieved using an initial internet survey with a pre-paid $5 cash incentive followed by a postal survey of non-responders [19]. Respondents to mail-only and internet+mail surveys were similar to each other in demographic characteristics and more closely matched population demographics than internet-only respondents. However, it was found that the mail-only comparison arms had higher response rates and lower costs, and the need to use postal contact for invitations and follow up negated the potential cost and speed advantages of the internet mode. In more restricted target group (subscribers to long distance telephone services), follow up of non-responders to four initial survey modes (mail, telephone, interactive voice response, or internet) using a different mode (telephone or mail) resulted in an increase of up to 37% in the overall response rate [20].

Investigation of factors affecting internet response rates among those who are successfully contacted shows general similarities to those documented for other survey modes [21]. The salience of the topic to the individual, the length and complexity of the survey and the type of sponsoring organisation (academic, government or commercial) all may influence a potential respondent’s decision to take part. Access to the internet is of course a prerequisite for responding, and the constraints of literacy and language apply for the internet mode as they do for mailed surveys, although the ability to provide access to questionnaires in alternative languages is greatly simplified on the internet.

### The effect of personalisation and incentives

Personalisation has generally been found to increase response rates for postal questionnaires, but a recent systematic review showed a positive effect in only one of six randomised studies that used community-based sampling frames [22]. Another analysis of 17 comparisons involving the general public in the US found a modest benefit of personalisation on mail survey response rates with greater effects apparent in rural areas [23]. Personalisation of email invitations for internet surveys has been reported to increase response rates in a number of groups including university students [24,25] and scientists and engineers [26]. This approach is not open to community surveys due to the lack of email lists, but personalisation of invitation letters for internet surveys might reasonably be expected to improve response rates compared to generic invitations.

Incentives for participation in surveys may be provided in monetary or non-monetary form, and paid unconditionally (sent to all potential respondents with the invitation), conditionally (sent only to those who complete the survey), or offered in the form of a lottery. Monetary incentives were shown to be effective in a systematic review of 94 postal surveys [22], with the odds of response being almost doubled compared to no incentive, although results were heterogenous. Monetary incentives were more effective than non-monetary incentives, and larger amounts generally more effective than smaller amounts. Unconditional incentives were more effective than conditional incentives, but there was heterogeneity among studies. In this review only one internet survey that assessed a monetary incentive versus no incentive was included, and there was no significant effect on the response rate. However six trials assessing non-monetary incentives found that the odds of response almost doubled compared to no incentive.

A variety of alternative or combined incentives have been assessed for internet surveys in terms of response rate and cost-effectiveness. For example, a Canadian study using mailed invitations for a community internet survey found a small ($2) prepaid cash incentive generated the highest response rate, but a high value lottery (2 prizes of $250) was the most cost-effective option (other options tested were no incentive and 10 prizes of $25) [27]. A systematic review of material incentives found a fairly consistent positive effect on increasing the number of people visiting the first page on internet survey websites but a more variable impact on the number of surveys actually completed [28], but most individual studies had insufficient power to detect an effect.

In 2011 we carried out a community-based survey in Melbourne, Australia examining household practices regarding greywater use and associated health risks. Greywater is used water collected from the bathroom, laundry or kitchen for subsequent reuse in the home or garden. We used postal invitations in combination with three modes of survey administration (postal, internet and telephone) and two styles of contact approach (personalised and generic), with a lottery incentive. We present a comparison of response rates and cost-effectiveness for the different modes and approaches.

## Methods

Ethical approval for the study was granted in compliance with the Helsinki Declaration by the Monash University Human Research Ethics Committee (MUHREC Project number CF10/1163-2010000621). Names and addresses for personalised contact were obtained from a commercial database holding the contact details of over 70% of Australian households and telephone numbers for some households. Five methods of recruitment were used:

1. Personalised postal survey: mail out of a personally addressed introductory letter, together with the explanatory statement for the study, a greywater survey form and a reply paid envelope.

2. Generic postal survey: mail out of an introductory letter addressed generically “to the householder”, together with the explanatory statement, a greywater survey form and a reply paid envelope.

3. Personalised internet survey: mail out of a personally addressed postcard inviting the householder to complete a web-based survey. The Explanatory statement was available at the web address given on the postcard. To prevent multiple survey responses from the same household, postcards were overprinted with a unique alphanumeric code which had to be entered to begin the survey. Each code could be used only once.

4. Generic internet survey: mail out of a generic postcard addressed “to the householder” inviting the householder to complete a web-based survey. These postcards also had a unique alphanumeric code.

5. Telephone interview: mail out of an explanatory statement and a personally addressed introductory letter informing the householder that they would be telephoned and invited to complete a telephone interview.

The total target number for completed surveys via all recruitment strategies combined was 600, with about 200 responses to be derived from each of the three modes of contact (postal, internet and telephone). The target area included approximately 128,000 residential postal delivery addresses in 27 contiguous suburbs in the southeast region of Melbourne. The selected area contained a high percentage of houses with gardens relative to the inner city, and therefore a higher likelihood of greywater use given the prevailing restrictions on outdoor tap water use imposed by prolonged drought conditions. The target area included suburbs spanning a range of socioeconomic levels defined by the Australian Bureau of Statistics (ABS) using Socio-Economic Indexes for Areas (SEIFA). The SEIFA Postal Area Index of Relative Socio-Economic Advantage and Disadvantage 2006 was used to categorise suburbs. This is a continuum of advantage (high values) to disadvantage (low values) and is derived from several Census variables, including factors such as tertiary education and household income [29].

The recruitment strategy ensured that each household received only one approach and this was accomplished by reserving two suburbs for the generic internet postcard mail out and one suburb for the generic postal survey mail out. For the remaining suburbs, separate non-overlapping data files were obtained for the personalised approaches (one data extract comprising 15,500 randomly selected names and addresses for the postal survey and another data extract containing 2,060 randomly selected names, addresses and telephone numbers for the telephone survey).

Printing, packaging and postal lodgement of survey material was carried out by a mailing company. Personalised postal surveys were sent to 5,500 householders, generic postal surveys to 4,000 householders, personalised internet postcards to 10,000 householders, generic internet postcards to 7,000 householders, and personalised letters informing householders they would be called for a telephone interview were sent to 2,060 households. Households selected for the telephone survey had a two week period to contact the Study Centre and decline participation before interviews began (a condition of MUHREC approval). Numbers listed in the Australian Do Not Call registry (a national opt-out list for telemarketing calls) were deleted prior to commencing telephone interviews. The generic postal surveys and internet postcards were delivered by the postal service to all households in the targeted suburb(s) along with conventional mail, rather than being bundled with “junk mail” deliveries. Householders had four weeks to respond to the postal and internet surveys, and no reminder mail outs or telephone calls were made. Telephone interviewing was contracted to a telemarketing company, and calls were made between 4 pm and 8 pm on Monday to Thursday of one week. Data entry for postal surveys was carried out in-house, and respondents entered their own data for the internet survey.

The survey enquired about collection and reuse of greywater (defined as water from the laundry, bathroom or kitchen) at any time in the last five years. For those who had used greywater, details were requested about specific greywater use practices. To enable demographic comparisons to be made, information was collected from all participants about postcode of residence, household type (family, couple, single, group), home ownership (own/purchasing, renting) and dwelling type (detached house, semi-detached house, apartment). The survey required a maximum of 10 min to complete, and the majority of questions required checkbox answers with some optional short text fields. Respondents to all survey modes (whether greywater users or non-users) could choose to enter a lottery draw for 20 retail gift vouchers to the value of $70 each.

A web database was established for data entry from all survey modes. The database programming included branching, so that questions deemed irrelevant based on a previous response were not presented to participants in the internet or telephone modes, minimising completion time. Programming also ensured that respondents could not skip relevant questions, thereby enhancing survey completeness and validity. Postal surveys included instructions for respondents to move to the next relevant section depending on their prior answers.

## Results

### Survey response rates

A total of 1677 responses to the greywater survey were received, comprising 924 postal surveys, 273 telephone surveys and 480 internet surveys. Among the postal surveys, 40 returned surveys were excluded from data entry due to large amounts of missing data. An additional 16 surveys (10 postal and 6 internet surveys) were excluded from data analysis as they were from respondents living outside the study area. This left 1621 valid surveys for analysis (874 postal, 273 telephone and 474 internet surveys), which was above the planned minimum number for each recruitment mode. Ninety seven (97) postal surveys lacked postcode information but were otherwise complete, and these were included in all analyses except those involving socioeconomic factors which required suburb of residence to be known. Overall, 67.6% of respondents were greywater users, and the majority of respondents (79.0%) opted to enter the prize draw.

Crude and adjusted response rates for the different modes of contact are shown in Table 1. The highest response rate was obtained for the telephone approach with 30.2% (95% confidence limit 27 – 33%) of households where at least one contact attempt was made, completing the survey. Contact modes which required the respondent to take action (fill in a postal survey form and mail it back, or visit an internet site to do an on-line survey) had lower response rates. For both postal and internet surveys, the personalised approach produced significantly higher crude response rates than the corresponding generic approach (two sample proportion test p < 0.001). After adjusting for mode characteristics (e.g. internet access, unknown postcode), the relative order of response rates remained the same as for crude rates: telephone survey personalised > postal survey personalised > postal survey generic > internet survey personalised > internet survey generic, with response rates significantly different from each other (two sample proportion test p < 0.001 in a stepwise manner).

**Table 1:** Response rates for the greywater use survey

**Type of approach**	**Number of approaches**	**Number of responses**	**Crude response rate (%)**	**Adjusted number of approaches**	**Adjusted number of responses**	**Adjusted response rate (%) and (95% CI)**
Postal survey personalised	5,500	511	9.3	5,500	575^*^	10.5 (9.7-11.3)
Postal survey generic	4,000	266	6.7	4,000	299^*^	7.5 (6.7-8.3)
Internet survey personalised	10,000	289	2.9	6,210^**^	289	4.7 (4.2-5.2)
Internet survey generic	7,000	101	1.4	4,515^***^	101	2.2 (1.8-2.6)
Telephone survey personalised	1,000	273	27.3	903^****^	273	30.2 (27.2-33.2)
Total	34,500	1621	4.7	25,384	1,621	6.4 (6.1-6.7)

^*^ Adjusted by distribution of 97 postal surveys with unknown postcode to personalised (n = 64) or generic modes (n = 33) as per survey response rates for known postcodes.

^**^ 62.1% of households in the personalised internet survey area had internet access.

^***^ 64.5% of households in the generic internet survey area had internet access.

^****^ Crude response rate calculated for 1,000 telephone numbers (minimum quantity for data file purchase), adjusted rate is for 903 telephone numbers with at least one contact attempt made. Of 866 households contacted, 352 refused, 148 were missed call backs, 93 had language difficulties. There were 37 numbers with technical difficulties.

As the generic postal surveys were sent to only two suburbs, the potential respondents may not have represented the same spectrum of socioeconomic categories included in the larger target area for the personalised postal survey. Therefore the response rate for the generic postal survey was also compared to the subset of suburbs in the same SEIFA quintile in the personalised approach area (quintile 4 for the SEIFA Postal Area Index of Relative Socio-Economic Advantage and Disadvantage 2006). A total of 794 personalised postal surveys had been sent to suburbs in this SEIFA quintile, resulting in 67 responses (crude response rate 8.4%) from the corresponding postcodes. This was higher than for the generic postal survey (crude response rate 6.7%), but the difference was not significant (p=0.09). For the personalised internet survey, the number of respondents from the corresponding SEIFA quintile area was too low to permit meaningful comparison with the generic internet survey area.

### Comparison of respondents from different survey modes

Information on participating households was compared with characteristics of residents of the target area using data from the 2006 Australian Census [30]. Survey respondents were more likely to be home owners/buyers (93.9% versus 72.9%), less likely to occupy apartments (1.2% versus 12.5%), and more likely to be families or couples (85.1% versus 74.5%) compared to the overall population in the target area.

Comparisons between survey respondents from different modes of contact showed that rates of home ownership were similar in all groups but telephone survey respondents included a significantly higher proportion of group households than postal or internet respondents (5.5% vs 0.8% and 1.9% respectively, p<0.01), and postal survey respondents were less likely to occupy apartments compared to telephone or internet respondents (0.6% versus 1.8% and 1.9% respectively, p<0.05). Greywater use was significantly lower among telephone respondents compared with both internet and postal respondents (both p<0.001) and for postal respondents compared with internet respondents (p<0.01).

### Comparison of respondents from different contact types

Comparison of respondents replying to generic versus personalised approaches showed no significant difference in household or dwelling type (both p>0.05) either overall or when adjusted for SEIFA quintile. However, those responding to generic approaches were less likely to be home owners/buyers than those who responded to personalised approaches (90.9% versus 95.0%, p=0.004). Additionally, there was a significantly higher proportion of greywater users among generic versus personalised approach respondents (75.6% versus 69.2%, p = 0.012). When the comparison was adjusted for SEIFA quintile, the differences were still significant.

### Costs of the survey

An assessment of the costs associated with each mode of survey recruitment and the expenditure required for each valid survey obtained is shown in Table 2. The estimated costs per completed valid survey were similar for the personalised ($24.75) and generic ($22.93) postal survey modes of recruitment, but costs for the internet and telephone survey modes were considerably higher, ranging from $44.24 to $53.84 per valid survey completed.

**Table 2:** Summary cost figures (Australian $) for the different survey modes

**Component**	**Postal survey personalised**	**Postal survey generic**	**Internet survey personalised**	**Internet survey generic**	Telephone survey^*^
Rental of data extract and data manipulation	$2,148	-	$3,817	-	$684
Printing and packaging survey form, letters, postcards	$4,625	$3,317	$2,494	$2,326	$509
Postage	$3,071	$683	$5,100	$966	$510
Web database programming^**^	$1,373	$1,373	$1,373	$1,373	$2,747
Salary costs for mail handling and data entry of postal surveys^***^	$3,012	$1,483	-	-	-
Telephone interviews	-	-	-	-	$10,249
**Total costs**	**$14,229**	**$6,856**	**$12,784**	**$4,665**	**$14,699**
Number of contact attempts	5,500	4,000	10,000	7,000	1,000
Number of valid surveys obtained	575	299	289	101	273
**Cost per valid survey**	**$24.75**	**$22.93**	**$44.24**	**$46.19**	**$53.84**

^*^ Costs have been calculated for 1,000 telephone numbers and introductory letters.

^**^ Web database programming and hosting costs have been distributed equally across postal, internet, and telephone survey modes.

^***^ Costs do not include salaries for senior researchers supervising staff.

## Discussion

Response rates for the different survey administration modes varied, as did greywater use rates among respondents to different modes, perhaps reflecting differing levels of motivation and interest in the topic. Similar to other community studies using postal invitations and comparing response rates for different survey modes [8,10], we found that response rates to the internet survey were significantly lower than other modes even after correction for levels of internet access. The crude response rates in our survey were significantly higher for personalised versus generic approaches with both postal and internet modes, but for the postal surveys there was no significant effect of personalisation when comparing areas of the same socioeconomic level, suggesting little if any benefit to overall response in this instance. However personalisation allows preliminary contact by letter prior to telephone interviews, and this has been shown to significantly increase response rates [31].

Our overall response rates were lower than has been documented in some other studies, but still enabled sufficient recruitment numbers for our purposes. This may in part reflect the specialised research topic, as well use of a single contact attempt with no follow up. Additionally, changing weather conditions (heavy rains before the survey period) and a relaxation in tap water restrictions in Melbourne probably reduced levels of interest in the survey topic. The response time permitted for postal and internet surveys was short (four weeks), and although we recognise that follow up of non-responders significantly enhances response rates to both postal [32] and internet surveys [6], we were unable to follow up non-respondents because of study time constraints. Similarly, the short time frame available for telephone interviews (four days) limited the ability to call back households who asked to defer the survey.

Our survey was not intended to assess the prevalence of greywater use in Melbourne but rather to collect detailed information on greywater use practices from households which had used this water source during a recent drought. Representative data on household greywater use in Australia are limited to the prevalence in state capitals and other areas, and garden watering as a major use [33]. For this reason it is difficult to ascertain whether our respondents are representative of all urban greywater users. The household characteristics of survey respondents were consistent with expectations, with greywater use being strongly associated with the presence of a garden and with home ownership. Our respondents spanned a range of socioeconomic levels, with no clear trend in response rates relative to socioeconomic status (data not shown). However, response required a good command of spoken or written English, and the effect of this restriction can be estimated from the telephone survey where 10.7% of contacted households were excluded because of language difficulties.

Analysis of costs per valid survey returned showed that the postal survey mode was the most cost-effective, with only minor differences between personalised and generic approaches, but internet and telephone modes were more costly. Our internet-based survey database was constructed by a professional programmer, and cost savings could be achieved by using a “do-it-yourself” web survey tool, provided that the survey structure is not too complex. However, costs need to be balanced against generalisability, and if the lower motivation required for telephone surveys means they are associated with increased response rates, they may also provide a more representative range of respondents than other survey modes.

The impact of changes in household telecommunications and other factors on the coverage and availability of different data sources mean that researchers must now consider use of more than one sampling frame and/or response mode in attempts to survey or recruit representative population samples [3,34]. Current evidence suggests that telephone and postal modes of survey administration remain the most effective for community surveys, but this may change as levels of household internet access and familiarity increase. There is also a need to develop more cost-effective ways of inviting community participation in internet surveys. Paid advertising on social media websites or pop-up adverts triggered by particular internet search terms have been successfully used for recruitment of specific target groups including young women [35] or people seeking quit smoking information [36], but it is not yet clear how such methods might be adapted to broader community recruitment efforts.

Regardless of the sampling frame or mode of contact, low response rates in comparison to historical levels seem likely to continue in the future. While high response rates have often been viewed as a safeguard against non-response bias, low response rates of themselves do not necessarily indicate a high degree of bias in study results. A detailed analysis of the relationship between non-response rates and non-response bias in 235 separate estimates from 30 published studies found that the response rate alone was not a very good indicator of the magnitude of non-response bias [37]. While very high response rates reduced the probability that non-response bias was present, this did not mean that the degree of bias was low, in cases when it did occur. In addition, there was often considerable variation in the degree of bias among different variables within any individual study.

## Conclusions

Our findings suggest that postal surveys remain the most economic option for community surveys, with similar costs for personalised and generic approaches. Telephone surveys may produce higher response rates but also entail higher costs, and the internet is not currently an effective mode for community-based surveys.

In Australia, as in many other jurisdictions, it is becoming increasingly difficult to obtain data sources which provide a representative population sampling frame for inviting community members to participate in epidemiological surveys. The optimal recruitment strategy for a specific study requires consideration of the relative importance of response rates, response biases, cost-effectiveness and generalisability. Even when a representative sampling frame is used, some degree of response bias is inevitable depending on demographic and socioeconomic factors, literacy and language constraints, the personal level of interest/relevance of the topic under study, the demands of the survey (length/complexity/sensitive topics), and the mode(s) of contact employed. When publishing study outcomes it is important that researchers specify the limitations of the data sources and sampling strategies used for recruitment, and document the potential biases in the target and respondent populations and how this may affect the data collected.

## Abbreviations

ABS: Australian Bureau of Statistics; RDD: Random digit dialling; SEIFA: Socio-economic indexes for areas.

## Competing interests

The authors declare that they have no competing interests.

## Authors’ contribution

All authors were involved in the study conception and design, interpretation of data, and reviewed drafts of the manuscript. JOT conducted the data analysis, MM and MS wrote the initial draft. All authors read and approved the final manuscript.

## Author information

MM is currently located at the Epidemiology Unit, Ministry of Health, Colombo, Sri Lanka.

## Pre-publication history

The pre-publication history for this paper can be accessed here:

http://www.biomedcentral.com/1471-2288/12/132/prepub
